# Liver Isolation Oxaliplatin (LIOX): Long Term Survival from a New Locoregional Technique for Chemorefractory Patients with Colorectal Liver Metastases

**DOI:** 10.1245/s10434-022-11348-z

**Published:** 2022-02-11

**Authors:** Nyan Y. Khin, Madhawa De Silva, Stephen Clarke, Nick Pavlakis, Chris M. Rogan, Kevin Ho-Shon, Rodney J. Lane

**Affiliations:** 1 School of Biomedical Engineering, University of Technology Sydney, Ultimo, Australia; 2 AllVascular Pty Ltd, St Leonards, NSW Australia; 3 Department of Medical Oncology, Royal North Shore Hospital, St Leonards, Australia; 4 Kolling Institute of Medical Research, St Leonards, Australia; 5 Royal Prince Alfred Hospital, Camperdown, Australia; 6 Sydney Adventist Hospital, Wahroonga, Australia; 7 Macquarie University Hospital, Macquarie Park, Australia; 8 Department of Vascular Surgery, North Shore Private Hospital, St Leonards, Australia

A ten-patient pilot study treating patients with unresectable liver metastases from colorectal adenocarcinomas, using a new locoregional technique known as repeated liver isolation oxaliplatin (LIOX), was completed between 2012 and 2015. The LIOX technique comprised of implanting a transcutaneous arterial access system on the patient’s axillary artery to facilitate simultaneous multicatheter access into the patient’s vasculature. Via the access system, balloon catheters were deployed in the patient’s coeliac trunk and superior mesenteric artery to indirectly obstruct the hepatic portal supply. The inferior mesenteric artery was tied off in this patient cohort. Another balloon catheter was then guided to the hepatic artery proper, the left/right branch, or a segmental artery for oxaliplatin infusion. On average, patients received five to six treatments with three catheters for each procedure over a 1-month period resulting in up to 18 cannulations made possible in a nonpercutaneous manner by the arterial access system. The patient demographics, workup, treatment details, safety and feasibility results, and clinical response have previously been reported.^1^ This correspondence is a follow-up to report overall survival (OS) data, post-study patient management data, as well as basic patient and treatment details, which are summarized in Table 1.

**Table 1:** Summary of patients’ LIOX treatment, clinical response, post-trial treatments, and overall survival

Patient	Sex/age	KRAS status	Prior lines of therapy	No. LIOX treatments	Treatment period (days)	Overall survival (mo)	∆CEA (%)^a^	Treatments after LIOX to end of survival		Clinical response
								Type	Details	
1	F/55	N/A	4+	3	14	5.1	− 72	–	None	Responsive
2	M/67	m	1	5	19	11.4	39	C R	FOLFIRI/bevacizumab Palliative scapula radiotherapy	Progressive
3	F/52	Wt	4+	6	28	12.9	− 79	S C	Attempted liver resection FOLFOX	Responsive
4	M/61	Wt	4+	8^b^	29	4.1	17	–	None	Progressive
5	F/60	m	2	4	35	68.1	0	C S C R C C C	Capecitabine/bevacizumab Interval resection of primary 5-FU/bevacizumab SBRT to liver FOLFIRI/bevacizumab Capecitabine/bevacizumab, Stat 3 inhibitor + nivolumab (trial) Trifluridine/tipiracil	Stable
6	M/66	Wt	4	5	28	4.7	− 8	R	Palliative pelvic radiotherapy	Progressive
7	M/67	N/A	2	6	28	35.9	67	C C	Capecitabine + bevacizumab FOLFOX	Stable
8	F/59	Wt	3	6	25	57.5	57	C R C C C C	Capecitabine/bevacizumab Yttrium Y-90 embolization FOLFIRI/cetuximab Trifluridine/tipiracil Capecitabine/mitomycin-C FOLFIRI/cetuximab	Responsive
9	M/51	Wt	4	7	24	10.8	− 23	C C	FOLFIRI/bevacizumab FOLRIFI/cetuximab	Progressive
10	M/69	m	3	7	30	7.0	51	R	Yttrium Y-90 embolization	Stable

*Wt* wild-type, *m* positive mutation, *N/A* not available, *C* chemotherapy, *R* radiotherapy, *S* surgery

^a^First reading available after final LIOX infusion minus last reading available prior first LIOX infusion

^b^Patient received eight HAI infusions instead of LIOX

**Fig. 1:**
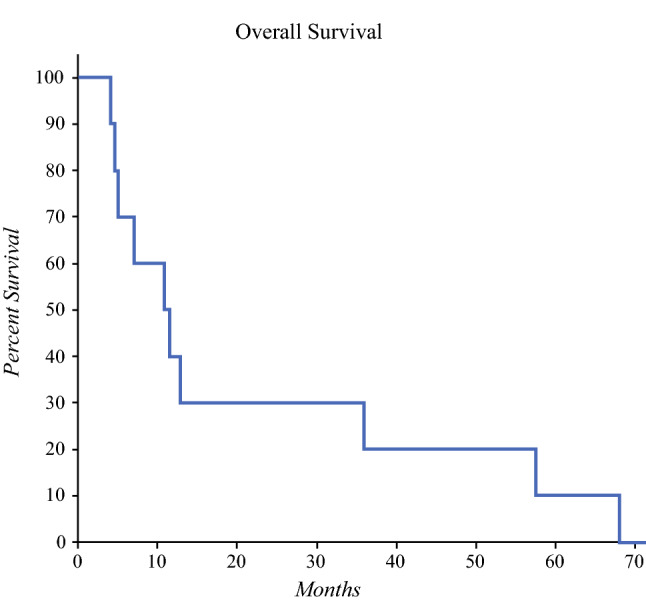
Kaplan-Meier graph of overall survival (OS) for entire patient cohort. Median OS was 11.1 months; 1-year, 2-year, 3-year, 4-year, and 5-year survival were 40%, 30%, 20%, 20%, and 10% respectively

The Kaplan–Meier graph for the ten patients in Fig. 1 shows a median OS of 11.1 (range 4.1–68.1) months. The survival data from this study, although not statistically powered, is comparable to that of what has been reported for newer agents, such as trifluridine/tipiracil (6.6–7.1 months)^2,3^ and regorafenib (6.4–9.3 months).^4,5^ Similarly, it is comparable to the median OS reported for locoregional therapies, such as yttrium-90 resin microspheres (Y^90^) when used alone (9.0 months),^6^ or in combination with systemic therapies (8.4–10.0 months).^7,8^

All but one patient had at least two lines of therapy before being enrolled on the LIOX study. It is worth noting that the patient with the shortest survival (4.1 months) received eight repeated hepatic arterial infusions (HAI) instead of LIOX^1^ due to a suboptimal angle of implantation of the arterial access system, which was corrected for all subsequent patients. Of note, the longest survivor (68.1 months) was a KRAS mutation-positive patient. There was an observable difference in the mean OS between patients with nonprogressive and progressive disease (31.1 vs 7.7 months; *P* = 0.07). There were no late vascular complications related to device implantation with longer follow-up. There was observable liver toxicity in the early posttreatment period (*n* = 7) characterized by asymptomatic twofold to fourfold increase in liver function tests. Six patients had evidence of deranged liver function in longer-term follow-up; however, interpretation is limited as this may be due to subsequent receipt of hepatotoxic systemic therapies or progressive disease. There were no significant late extrahepatic toxicities.

The LIOX treatment is a repeatable and more controlled approach to liver direct therapy compared with other locoregional treatments. The risks associated with radioembolization induced liver disease and hepatopulmonary shunting limit the repeatability of Y^90^ while clinicians must commit to either a whole-liver or lobar confined treatment with hepatic arterial infusion (HAI). The absence of radioembolic agents with LIOX enables repeatability which is a well-established contributor to efficacy as evidenced through systemic chemotherapy. LIOX also provides flexibility by allowing clinicians to alternate between whole liver, lobar, and selective intra-arterial chemotherapy without being prone to dilution and washout from the uncontrolled hepatic portal blood supply as is the case with Y^90^ and HAI.

Given that none of the patients in the pilot study were oxaliplatin-naïve, they would have been either unresponsive to their initial systemic oxaliplatin-based therapy or were responsive but had to cease treatment due to the onset of severe oxaliplatin related side-effects. The study’s use of oxaliplatin in a salvage and rechallenge setting in the form of LIOX is suggestive that the route of delivery may be consequential. LIOX may be an option for patients to complete the oxaliplatin component of a patient’s doublet/triplet chemotherapy regimen in patients who are responsive to the agent but unable to tolerate its cytotoxic side-effects. The pilot study demonstrated feasibility of LIOX with comparable outcomes to newer agents and locoregional treatments used for refractory colorectal liver metastases with no major late toxicity signals. A follow-up Phase Ib/II study (NCT04701281) is currently underway to treat patients with refractory disease or patients with a RAS-positive mutation midway into their first line therapy. LIOX is a novel locoregional technique that could be used in conjunction with any therapeutic agent, and as such, presents an opportunity to reassess the full potential of existing agents proven to be effective in the intravenous setting such as oxaliplatin, 5-FU, FUDR, and irinotecan.
